# The Effect of Footwear, Running Speed, and Location on the Validity of Two Commercially Available Inertial Measurement Units During Running

**DOI:** 10.3389/fspor.2021.643385

**Published:** 2021-04-26

**Authors:** Christopher Napier, Richard W. Willy, Brett C. Hannigan, Ryan McCann, Carlo Menon

**Affiliations:** ^1^Menrva Research Group, Schools of Mechatronic Systems Engineering and Engineering Science, Simon Fraser University, Metro Vancouver, BC, Canada; ^2^Department of Physical Therapy, University of British Columbia, Vancouver, BC, Canada; ^3^School of Physical Therapy and Rehabilitation Science, University of Montana, Missoula, MT, United States; ^4^Biomedical and Mobile Health Technology Laboratory, Department of Health Sciences and Technology, ETH Zurich, Switzerland

**Keywords:** running, biomechanics, validity, inertial measurement units, footwear

## Abstract

**Introduction:** Most running-related injuries are believed to be caused by abrupt changes in training load, compounded by biomechanical movement patterns. Wearable technology has made it possible for runners to quantify biomechanical loads (e.g., peak positive acceleration; PPA) using commercially available inertial measurement units (IMUs). However, few devices have established criterion validity. The aim of this study was to assess the validity of two commercially available IMUs during running. Secondary aims were to determine the effect of footwear, running speed, and IMU location on PPA.

**Materials and Methods:** Healthy runners underwent a biomechanical running analysis on an instrumented treadmill. Participants ran at their preferred speed in three footwear conditions (neutral, minimalist, and maximalist), and at three speeds (preferred, +10%, −10%) in the neutral running shoes. Four IMUs were affixed at the distal tibia (IMeasureU-Tibia), shoelaces (RunScribe and IMeasureU-Shoe), and insole (Plantiga) of the right shoe. Pearson correlations were calculated for average vertical loading rate (AVLR) and PPA at each IMU location.

**Results:** The AVLR had a high positive association with PPA (IMeasureU-Tibia) in the neutral and maximalist (*r* = 0.70–0.72; *p* ≤ 0.001) shoes and in all running speed conditions (*r* = 0.71–0.83; *p* ≤ 0.001), but low positive association in the minimalist (*r* = 0.47; *p* < 0.05) footwear condition. Conversely, the relationship between AVLR and PPA (Plantiga) was high in the minimalist (*r* = 0.75; *p* ≤ 0.001) condition and moderate in the neutral (*r* = 0.50; *p* < 0.05) and maximalist (*r* = 0.57; *p* < 0.01) footwear. The RunScribe metrics demonstrated low to moderate positive associations (*r* = 0.40–0.62; *p* < 0.05) with AVLR across most footwear and speed conditions.

**Discussion:** Our findings indicate that the commercially available Plantiga IMU is comparable to a tibia-mounted IMU when acting as a surrogate for AVLR. However, these results vary between different levels of footwear and running speeds. The shoe-mounted RunScribe IMU exhibited slightly lower positive associations with AVLR. In general, the relationship with AVLR improved for the RunScribe sensor at slower speeds and improved for the Plantiga and tibia-mounted IMeasureU sensors at faster speeds.

## Introduction

Running is one of the most popular leisure-time physical activities worldwide owing to its accessibility and low cost. Recreational running is growing in participation (Scheerder et al., 2015; Bush, 2017)—an effect that has been amplified by the closure of gyms and community centers during the COVID-19 pandemic (Minsberg, 2020; Ronto, 2020). Most running-related injuries are believed to be caused by an abrupt change in training load (Hreljac, 2005; Bertelsen et al., 2017; Napier, 2020), compounded by biomechanical movement patterns (Ryan et al., 2006; Napier et al., 2018; Ceyssens et al., 2019; Napier, 2020). Wearable technology allows the collection of both biomechanical and training load data longitudinally in the runner's natural environment (Napier et al., 2017; Willy, 2018; Moore and Willy, 2019). Advances in technology have made it possible to fuse biomechanical and training load measures to better quantify the cumulative stress (i.e., the additive stress of repeated steps during a run or repeated running bouts over the course of a training block) on the body, with much recent work utilizing segmental accelerations as a quantification of biomechanical training loads (Napier et al., 2020; Paquette et al., 2020).

The most ubiquitous class of wearable sensors is the inertial measurement unit (IMU), which consists of an accelerometer, gyroscope, and (sometimes) a magnetometer to measure accelerations, angular velocities, and orientation, respectively. Due to their ease of use and potential to assess biomechanical training loads related to running-related injuries, tibial accelerometers are commonly used in the study of running-related injury prevention or rehabilitation (Willy, 2018; Moore and Willy, 2019). Vertical peak positive acceleration (PPA)—the maximum acceleration in the vertical axis—is typically measured at the distal tibia. Vertical PPA of the tibia has been associated with running-related injuries (e.g., tibial stress fracture) (Milner et al., 2006; Pohl et al., 2008) and has been associated with the vertical ground reaction force (GRF) loading rate (Hennig and Lafortune, 1991; Laughton et al., 2003; Tenforde et al., 2020). For an IMU to capture PPA during running, it needs to sample data at a high enough frequency while having a dynamic range of at least 16 gravitational equivalents (g) (Mitschke et al., 2017; Willy, 2018). Research-grade IMUs are now affordable to clinicians and consumers, but few devices have established criterion validity (Willy, 2018; Moore and Willy, 2019).

While PPA measured at the distal tibia has demonstrated good validity and reliability in research settings (Sheerin et al., 2018), IMUs must also be easily and securely fixated to be user-friendly at the clinical or consumer level. A tibia-mounted IMU requires a consistent and secure mounting to the distal tibia prior to each use, which may affect reliability of impact-related metrics (Sheerin et al., 2019). An unreliable signal would in turn reduce the validity of the PPA metric derived from the tibia-mounted IMU as a surrogate for vertical GRF loading rates. As such, consumer-level IMUs are often mounted on the shoe (e.g., RunScribe, Stryd, Garmin FootPod). Shoe-mounted IMUs typically provide higher peak acceleration values than those mounted on the distal tibia (Cheung et al., 2019; Sheerin et al., 2019). Distally-placed accelerometers may also more closely represent the accelerations experienced by the foot/ankle (Sheerin et al., 2019). However, positive associations between impact loading (average vertical loading rate; AVLR) and PPA of shoe-mounted IMUs have been poor, especially when attached to the heel of the shoe (Cheung et al., 2019; Pairot de Fontenay et al., 2020).

The RunScribe sensor (Scribe Labs Inc., San Francisco, USA) is a commercially available IMU that has been validated for several spatiotemporal and kinematic metrics (Koldenhoven and Hertel, 2018; García-Pinillos et al., 2019; Hollis et al., 2019). When mounted on the shin adjacent to a research-grade accelerometer, the RunScribe sensor also demonstrated high positive associations (ICC 0.89–0.92) with the measurement of tibial PPA across a range of running speeds (Brayne et al., 2018). The RunScribe sensor mounted on the heel is not a valid surrogate for either the average or instantaneous vertical loading rates (Pairot de Fontenay et al., 2020), indicating that choice of sensor location can have important implications. However, current guidelines from RunScribe recommend placing it on the dorsum of the shoe, where it clips into a cradle that is securely mounted to the shoelaces. To our knowledge, the validity or reliability of impact metrics for this device at this location have not yet been investigated.

Plantiga (Plantiga Technologies, Vancouver, Canada) is another commercially available IMU that has not been validated. This IMU is embedded in the heel of an insole in place of a standard running shoe insole. The location of this sensor has two main advantages: (1) the device is easily and consistently fixated to its location and (2) the location is at the interface of the foot and the shoe, enabling it to capture the initial shock of impact during a rearfoot strike. To our knowledge, to date, there have been no studies examining the validity or reliability of this device.

In addition to sensor location, running speed (Sinclair et al., 2013b; Boey et al., 2017; Sheerin et al., 2018) and the footwear worn (Sinclair et al., 2013a,b, 2016; Sinclair and Sant, 2017) are also known to affect impact-related metrics. Running at greater speeds or in minimalist shoes has been consistently associated with increased PPA when measured at the tibia (Sheerin et al., 2019). Running speed and footwear also affect vertical GRF measures. The AVLR increases with greater running speeds (Napier et al., 2019) while minimalist footwear tends to increase vertical loading rates when compared to cushioned footwear (Moore et al., 2015; Warne et al., 2017). Therefore, any investigation into the relationship between PPA and vertical GRF loading rates should also consider these factors.

The aim of this study was to assess the validity of two commercially available IMUs (RunScribe and Plantiga) during running for the measurement of vertical peak positive acceleration. The gold standard measure for comparison was AVLR because of its common use in lab-based running injury studies. A second comparison was made to a research-grade tibia-mounted IMU (IMeasureU Blue Thunder, Vicon, Oxford, UK) since vertical (or axial) PPA is commonly used as a surrogate for AVLR. Secondary aims were to determine the effect of footwear, running speed, and the location of the IMU on the vertical peak positive acceleration. We compared the consumer-grade IMUs to research-grade tibia- and shoe-mounted IMUs and the average vertical loading rate measured on a force treadmill. We hypothesized that the RunScribe IMU would not display a strong association with GRF or tibia-mounted IMU measures, but that the insole-embedded Plantiga IMU would be. We also hypothesized that the vertical PPA would be significantly less for the tibia-mounted IMU than the RunScribe and Plantiga IMUs, and that PPA would be greater in minimalist shoes and at greater speeds, since both have been shown to affect PPA measurement.

## Materials and Methods

### Participants

Healthy runners between the ages of 18 and 60, free of musculoskeletal and neurological pain, and who had been running for at least 3 months were recruited from the local running community. Participants were excluded if they were not habitual rearfoot strikers or did not fit the range of shoe sizes available for the study (Men's 8–12 US or Women's 6–10 US). Participants were screened for inclusion/exclusion criteria *via* an eligibility questionnaire. Habitual foot strike pattern was self-reported and confirmed during the warmup period before data was collected. Written consent was obtained from all participants and ethics approval was granted from the institutional Clinical Research Ethics Board.

### Experimental Protocol/Procedures

**Figure 1:**
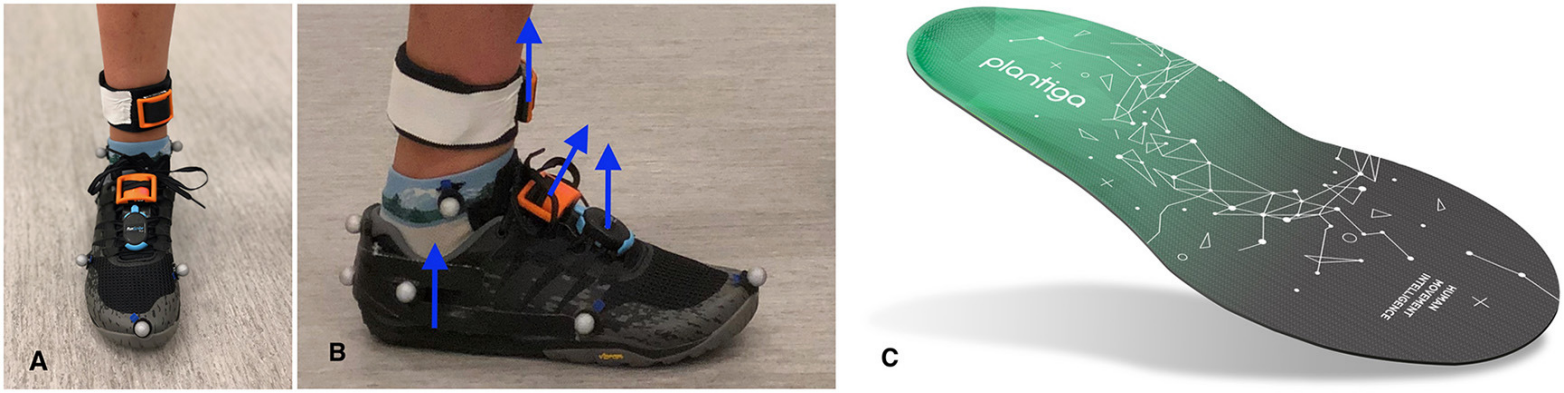
**(A)** Set up for the IMeasureU (tibia and shoe-mounted) and RunScribe sensors; **(B)** “vertical” axes for each IMU illustrated by location; and **(C)** Plantiga insole-embedded inertial measurement unit.

All participants underwent a biomechanical running analysis on an instrumented treadmill (Bertec Corporation, Columbus, USA) wearing standardized running shoes (Neutral: New Balance 880v9, New Balance, Boston, USA; Minimalist: Merrell Trail Glove 5, Merrell, Grand Rapids, USA; Maximalist: New Balance Fresh Foam More v1, New Balance, Boston, USA). A preferred speed representative of a moderate intensity run was determined during an initial 5-min. warmup period. Participants then ran at their preferred speed in each footwear condition (NEUT: neutral; MIN: minimalist; MAX: maximalist) and at preferred speed + 10% (NEUT +10%), and preferred speed −10% (NEUT −10%) in the neutral running shoes. The order of footwear was randomized, with the order of speed in the NEUT condition proceeding from preferred speed to NEUT +10% to NEUT −10%. Each trial consisted of approximately 1 min of running. Four wearable sensors were fixed at the distal tibia (IMeasureU-Tibia), shoelaces (IMeasureU-Shoe and Runscribe), and insole (Plantiga) of the right shoe, as shown in Figure 1 and data were collected during quiet standing to enable calibration of the IMeasureU and Plantiga devices during post-processing. With this configuration, the IMeasureU sensor was positioned so that the positive *Y*-axis and the positive *Z*-axis were vertical on the distal tibia and shoe, respectively. The specifications of each device are detailed in Table 1. Kinetic data from the treadmill were sampled at 2,000 Hz (Cortex v5, Motion Analysis Corporation, Santa Rosa, USA). Accelerometer data from the IMUs were sampled at 1,000 Hz (IMeasureU) and 500 Hz (Plantiga and RunScribe). IMUs were started and stopped manually. Every effort was made to stop the IMU devices within 1–2 s of the end of each trial as marked by the GRF data capture. As such, the steps were not perfectly synchronized, but the trials were temporally proximal to each other.

**Table 1:** Specifications of inertial measurement units.

	**Location**	**Weight**	**Sampling frequency**	**Dynamic range**
Blue Thunder, IMeasureU	Tibia, laces	12 g	1,000 Hz	± 16 g
Plantiga	Insole	17.5 g	500 Hz	± 16 g
RunScribe	Laces	15 g	500 Hz	± 16 g

### Data Analysis

**Figure 2:**
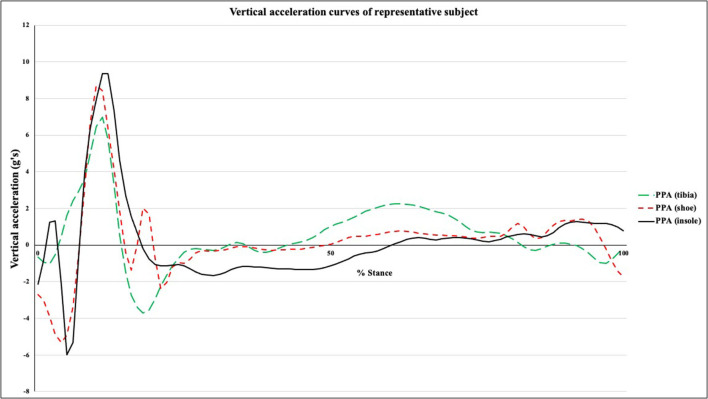
Vertical acceleration profile of tibia, shoe-mounted, and insole-embedded inertial measurement units.

Kinetic variables were calculated using The MotionMonitor software (Innovative Sports Training, Inc., Chicago, USA). CSV files were exported from the IMUs and Plantiga devices for signal processing. Discrete variables and accelerometry data were analyzed using custom LabView software (Version 17.0, National Instruments, Houston, USA) for the last 30 consecutive steps of the right foot for each of the five trials (NEUT, MIN, MAX, NEUT +10%, NEUT −10%). Force plate and kinematic data were low-pass filtered *via* a low-pass, fourth-order Butterworth recursive filter at a cutoff frequency of 50 and 15 Hz, respectively. Initial contact and toe-off events in the force plate signal were identified by a vertical GRF threshold of 50 N. The primary outcome from the GRF data was average vertical loading rate (AVLR). Since true impact frequencies range from 40 to 60 Hz (Valiant et al., 1987; Winslow and Shorten, 1989), a cut-off frequency of 75 Hz was used to ensure that only non-physiological frequencies were removed from the accelerometry signal (Crowell and Davis, 2011). Accelerometer data from the IMeasureU and Plantiga devices were filtered *via* a low-pass, fourth-order Butterworth recursive filter at a cutoff frequency of 75 Hz. *Via* the customized LabView software, accelerometry signals underwent a post-collection calibration process during which any signal offset and drift was removed prior to movement trials (Winslow and Shorten, 1989). Footstrikes from accelerometry data from the IMeasureU and Plantiga devices were identified at 0.1 ms prior to a maximum of the vertical accelerometer signal (Johnson et al., 2020). Primary IMU outcome variables were PPA from the IMeasureU-Tibia and IMeasureU-Shoe; PPA from the Plantiga IMU (Figure 2); and proprietary algorithms for “Impact” and “Shock” from the RunScribe device. “Impact” is equivalent to vertical PPA, while “Shock” represents the resultant PPA (vector sum of the XYZ acceleration components) from the RunScribe sensor.

### Statistical Analysis

An a priori sample size calculation for an expected Pearson product-moment correlation coefficient of 0.65 (*p* < 0.05, ß = 0.90) between AVLR and the consumer-grade IMUs produced a necessary sample size of 17 participants. Normality of all variables was assessed using Shapiro-Wilk tests. Scatterplots were checked for non-linear relationships and outliers. Pearson product-moment correlations (*r*) were calculated for the mean values of AVLR and PPA for each participant over the last 30 consecutive steps. Associations were classified as low (0.30–0.49), moderate (0.50–0.69), high (0.70–0.89), and very high (0.90–1.0) (Hinkle et al., 2003). Moderate to very high correlations were considered to be clinically meaningful. Further interpretation of the associations between PPA from each IMU and the primary GRF outcome (AVLR) was provided using the coefficient of determination (*r*^2^), which estimates explained variation in the dependent variable. Repeated measures ANOVA with *post hoc* comparisons using Bonferroni adjustment were performed to investigate differences in impact measures between speeds and footwear. Paired *t*-tests were used to determine differences in PPA between IMU locations. Significance for all statistical tests was set to *p* < 0.05 with trends identified between 0.05 ≤ *p* ≤ 0.10. All statistical analyses were performed using IBM SPSS Statistics for Mac, version 27.0 (IBM Corp., Armonk, USA).

## Results

Twenty participants (nine females; age 35.9 ± 8.3 years; BMI 21.1 ± 2.7 kg/m^2^; preferred speed 2.90 ± 0.37 m/s) participated in the study. Due to data collection errors, not all trials were captured for each measure. Specifically, out of a possible total of 100 participant trials, one participant was missing GRF data (failed to zero force plates before trial); 11 trials (across three participants) were missing data due to a faulty Plantiga sensor; and 10 trials (across three participants) were missing data from the IMeasureU-Tibia and one participant was missing data from the IMeasureU-Shoe due to collection errors (e.g., manual data recording not started on a device). All variables were found to be normally distributed (*p* > 0.05) with linear relationships and no outliers. The mean values for the GRF and IMU measures are reported in Table 2.

**Table 2:** Values (mean ± SD) for vertical ground reaction force and inertial measurement unit measures across all trials.

	***Overall (SEM)***	**NEUT**	**MIN**	**MAX**	**NEUT +10%**	**NEUT –10%**
AVLR (BW/s)	72.89 ± 22.17(2.27)	67.24 ± 17.79^a^^b^	91.39 ± 25.67^y^	70.00 ± 18.29^x^	77.23 ± 19.98^b^	58.58 ± 14.71^a^
Plantiga (g)	7.55 ± 2.31(0.25)	7.24 ± 2.01^x^	8.85 ± 2.11^∧^	7.23 ± 2.84	7.67 ± 2.25	6.76 ± 1.95
IMeasureU-Tibia (g)	7.32 ± 2.84(0.30)	7.17 ± 2.71^b^	7.78 ± 2.41	8.18 ± 3.48	7.57 ± 2.98^b^	5.92 ± 2.31^a^
IMeasureU-Shoe (g)	7.14 ± 2.88(0.30)	7.15 ± 3.06	7.22 ± 2.62	6.89 ± 3.13	7.93 ± 3.00^b^	6.53 ± 2.69^a^
RunScribe Shock (g)	14.15 ± 2.21^‡^(0.22)	13.87 ± 2.34^a^^b^^‡^	15.35 ± 1.17^‡^	14.02 ± 2.15^‡^	14.71 ± 2.20^b^^‡^	12.82 ± 2.33^a^^‡^
RunScribe Impact (g)	12.15 ± 2.46^‡^(0.25)	11.87 ± 2.62^‡^	13.37 ± 1.73^‡^	12.08 ± 2.36^‡^	12.56 ± 2.39^b^^‡^	10.89 ± 2.62^a^^‡^

a *Significantly different (p < 0.05) from NEUT +10% across same sensor*;

b *Significantly different (p < 0.05) from NEUT−10% across same sensor*;

x *significantly different (p < 0.05) from MIN across same sensor*;

y *significantly different (p < 0.05) from MAX across same sensor*;

∧ *significantly different from PPA (shoe) across same condition*;

‡ *significantly different (p < 0.05) from all other IMU measures across same condition. SEM, standard error of the mean; NEUT, neutral shoes at preferred speed; MIN, minimalist shoes at preferred speed; MAX, maximalist shoes at preferred speed; NEUT +10%, neutral shoes at 10% faster than preferred speed; NEUT −10%, neutral shoes at 10% slower than preferred speed; AVLR, average vertical loading rate; PPA, peak positive vertical acceleration; BW/s, body weights per second; g, gravitational forces*.

Significant Pearson product-moment correlations were found between all outcomes when all trials were included (Table 3). The AVLR exhibited high positive associations with IMeasureU-Tibia in the NEUT and MAX shoes and in all running speed conditions, but had only low positive association in the MIN footwear condition. Conversely, associations between AVLR and Plantiga were high in the MIN condition and moderate in the NEUT and MAX footwear, with low and moderate positive associations, respectively, in the NEUT −10% and NEUT −10% running speed conditions. The RunScribe Impact and Shock metrics demonstrated low to moderate positive associations with AVLR across all footwear and speed conditions with the exception of non-significant correlations for Impact in the MIN condition and for Shock in the NEUT +10% condition. In general, the relationship with AVLR improved for the shoe-mounted (RunScribe and IMeasureU-Shoe) sensors at slower speeds and improved for the Plantiga and IMeasureU-Tibia sensors at faster speeds. Overall, 41% of the variance in the value of AVLR was explained by variations in IMeasureu-Tibia, while 37% was explained by variations in Plantiga and 30% by RunScribe Impact. At the faster speed in the neutral shoe, variations in IMeasureU-Tibia explained up to 68% of the variance in the value of AVLR. Variations in Plantiga explained up to 56% of the variance in AVLR in the minimalist shoe and variations in Impact explained up to 38% of the variance in AVLR in the neutral shoe at preferred speed.

**Table 3:** Pearson product-moment correlations among vertical ground reaction force (average vertical loading rate, AVLR) and inertial measurement unit (peak positive vertical acceleration, PPA) measures across all conditions.

	**Overall**	**NEUT**	**MIN**	**MAX**	**NEUT +10%**	**NEUT –10%**
AVLR—Plantiga	0.61^‡^	0.50^*^	0.75^‡^	0.57^†^	0.53^*^	0.44^*^
AVLR—IMeasureU-Tibia	0.64^‡^	0.72^‡^	0.47^*^	0.70^‡^	0.83^‡^	0.71^‡^
AVLR—IMeasureU-Shoe	0.49^‡^	0.51^*^	0.45^*^	0.46^*^	0.58^†^	0.75^‡^
AVLR—RunScribe Shock	0.47^‡^	0.43^*^	0.40^*^	0.50^*^	0.20	0.40^*^
AVLR—RunScribe Impact	0.55^‡^	0.62^†^	0.38	0.58^†^	0.40^*^	0.50^*^
IMeasureU-Tibia—Plantiga	0.47^‡^	0.47^*^	0.19	0.60^†^	0.55^*^	0.43
IMeasureU-Tibia—IMeasureU-Shoe	0.72^‡^	0.68^‡^	0.53^†^	0.81^‡^	0.80^‡^	0.82^‡^
IMeasureU-Tibia—RunScribe Shock	0.42^‡^	0.34	−0.29	0.54^*^	0.40	0.60^†^
IMeasureU-Tibia —RunScribe Impact	0.46^‡^	0.43^*^	−0.28	0.59^†^	0.55^†^	0.66^†^
IMeasureU-Shoe—RunScribe Shock	0.57^‡^	0.70^‡^	0.36	0.48^*^	0.56^†^	0.71^‡^
IMeasureU-Shoe—RunScribe Impact	0.63^‡^	0.75^‡^	0.45^*^	0.53^*^	0.67^‡^	0.74^‡^

* *Indicates p < 0.05*;

† *p < 0.01*;

‡ *p ≤ 0.001. NEUT, neutral shoes at preferred speed; MIN, minimalist shoes at preferred speed; MAX, maximalist shoes at preferred speed; NEUT +10%, neutral shoes at 10% faster than preferred speed; NEUT −10%, neutral shoes at 10% slower than preferred speed; AVLR, average vertical loading rate; PPA, peak positive vertical acceleration*.

Running speed had a significant effect on AVLR and all PPA measures except the Plantiga (Table 2), with lower values at the slower speeds and higher values at the faster speeds. Footwear also influenced impact metrics with AVLR being significantly greater in the MIN (91.39 ± 25.67 BW/s) condition compared to the MAX (70.00 ± 18.29 BW/s; *p* = 0.023) condition and the Plantiga being significantly greater in the MIN (8.85 ± 2.11 g) compared to NEUT (7.24 ± 2.01; *p* = 0.025) condition. However, there were no significant differences between footwear conditions among the other IMU measures.

Across all footwear conditions and running speeds, RunScribe Impact and Shock were the only metrics to demonstrate statistically significant differences from all other IMU locations. However, the Plantiga (8.85 ± 2.11 g) was significantly different from the shoe-mounted metrics (IMeasureU-Shoe: 7.22 ± 2.62 g, *p* = 0.013; RunScribe Impact: 13.37 ± 1.73 g, *p* < 0.001; and Shock: 15.35 ± 1.17 g, *p* < 0.001) in the MIN footwear condition.

## Discussion

In partial support of our primary hypothesis, the insole-embedded IMU (Plantiga) demonstrated a stronger association with vertical GRF loading rate measures than the shoe-mounted IMU (RunScribe). The Plantiga had stronger associations with AVLR at faster running speeds and in minimalist footwear. In contrast, the RunScribe had stronger associations with AVLR at slower running speeds and in the more cushioned footwear conditions. However, both the RunScribe and Plantiga IMUs exhibited a low-positive association with the IMeasureU-Tibia overall, and this relationship varied across both footwear and speed conditions. One major concern with shoe-mounted IMUs is the excessive noise that can accompany the signal due to poor fixation and an uncoupling from the body (Cheung et al., 2019). Faster running speeds and minimalist footwear increase peak vertical accelerations at impact (Sheerin et al., 2019), which is likely to produce more movement artifact and increased signal noise in poorly fixated devices. This could explain the poorer performance of the shoe-mounted sensor at faster running speeds and in minimalist footwear. Previous studies have reported a range of correlations between tibia-mounted PPA and AVLR (*r* = 0.47–0.82) (Hennig et al., 1993; Laughton et al., 2003; Van den Berghe et al., 2019; Tenforde et al., 2020). Our results (*r* = 0.44–0.75 across all conditions) are consistent with these previous studies and support previous findings that values measured at the tibia increase with running speed (Sinclair et al., 2013b; Boey et al., 2017; Sheerin et al., 2018).

Our secondary hypotheses that the vertical PPA would be significantly less for the IMeasureU-Tibia than the RunScribe and Plantiga IMUs, and that PPA would be greater in minimalist shoes and at greater speeds, was also partially supported. Consistent with previous studies (Sheerin et al., 2019), the PPA was greater for all measures except the Plantiga at faster running speeds. The AVLR values were also significantly higher across faster speeds. Footwear also had the expected effect on vertical GRF loading rate measures, which showed a statistically significant increase in the AVLR in the minimalist shoe when compared to the maximalist shoe. This finding is in agreement with previous studies that reported substantially greater vertical loading rates in minimalist shoes compared to neutral cushioned shoes (Moore et al., 2015; Warne et al., 2017). The Plantiga IMU also demonstrated a significantly higher PPA in the minimalist shoes compared to neutral shoes. Significant effects were not seen in other PPA measures, possibly due to the more remote location from the foot-midsole cushioning-ground interface.

We also hypothesized that the location of the IMU would affect the magnitude of the PPA, with greater magnitudes occurring at the shoe-mounted and insole-embedded IMUs when compared to the more proximally located tibia-mounted IMU. Location (proximal to distal), vibration, sampling frequency, dynamic range, and sensor size can influence the magnitude of PPA reported by an IMU (Norris et al., 2014; Mitschke et al., 2017; Sheerin et al., 2019). However, the only significantly different signals from all of the IMUs in our analysis were the Impact and Shock metrics from the RunScribe sensor. These measures were significantly different from all other IMUs across all conditions. While the mean PPA values from the IMeasureU-Tibia, IMeasureU-Shoe, and Plantiga sensors were very similar, despite their differences in location, the Impact and Shock metrics from the RunScribe sensor were 1.5–2 times greater. One potential reason for this difference was the more distal location on the shoe of the RunScribe sensor (see Figure 1). During *post hoc* testing, it was noted that an IMU placed more distally on the dorsum of the shoe regularly produced greater peak accelerations than the more proximal location. Another potential reason for greater values among the RunScribe metrics is the proprietary algorithm used to calculate these variables since the RunScribe app does not filter the acceleration data. Normally, signal filtering would be expected to attenuate the accelerometry signal magnitude. Another possibility for the difference between the RunScribe sensor metrics and other IMU measures (notably the IMeasureU sensor that was mounted adjacent to it on the laces) could be the orientation of the device and how this was calculated into the output. The vertical acceleration signal from the IMeasureU-Shoe sensor was perpendicular to the sensor and not to the anterior-posterior axis of the shoe whereas the RunScribe sensor underwent a calibration procedure—as recommended by the manufacturer—before each test once it was mounted on the shoe, which transforms the signal from the sensor to the shoe coordinate frame (i.e., the vertical direction is perpendicular to the ground in standing). The difference between these axes is illustrated in Figure 1B.

Overall mean values in this study were comparable to previous studies for AVLR (Napier et al., 2018; Van den Berghe et al., 2019) and tibia PPA (Hennig et al., 1993; Creaby and Franettovich Smith, 2016; Van den Berghe et al., 2019; Tenforde et al., 2020) for similar running populations and speeds. Since this is the first investigation, to our knowledge, of PPA derived from lace-mounted or insole-embedded IMUs, we are unable to make similar comparisons across the literature. One recent study reported PPA values at the shoe (heel-mounted IMU) up to four times the magnitude (~ 9–14 g) of those at the tibia (Cheung et al., 2019). However, the authors noted that this difference was unlikely only due to the attenuation of forces across the foot and ankle, and suggested that the PPA at the shoe was amplified by the uncoupled shoe movements with respect to the body.

The relationship between AVLR and PPA from the shoe-mounted IMUs (IMeasureU-Shoe: *r* = 0.49 overall; RunScribe Impact: *r* = 0.55 overall; RunScribe Shock: *r* = 0.47 overall) was not only significant, but higher than in previous studies that fixed the IMU to the heel instead of the laces (Cheung et al., 2019; Pairot de Fontenay et al., 2020). This suggests that the lace-mount might be a better location to act as a proxy for GRF loading rates when compared with the heel. However, correlations for both of these sensors were still lower than with the IMeasureU-Tibia and Plantiga IMUs. Furthermore, while the IMeasureU-Shoe sensor had a high positive association with the IMeasureU-Tibia (*r* = 0.72), the RunScribe Impact metric only had a moderate positive association (*r* = 0.46) suggesting that this device in particular should not be used as a proxy for tibia PPA. This discrepancy could have been due to the lower sampling frequency of the RunScribe sensor which can result in an inaccurate reading of PPA (Norris et al., 2014; Mitschke et al., 2017). Overall, the association between the two shoe-mounted sensors was moderate (IMeasureU-Shoe—RunScribe Impact: *r* = 0.63) and high in the neutral shoes at preferred and −10% speeds (*r* = 0.75 and 0.74, respectively), suggesting that movement artifact for these sensors might have played a role in reducing the reliability of the signal with higher impacts at faster speeds and in minimalist shoes. However, the PPA values from the two sensors were significantly different from each other in all conditions.

The insole-embedded IMU from Plantiga had a stronger relationship with AVLR overall, which was comparable to the AVLR—IMeasureU-Tibia relationship (*r* = 0.61 and 0.64, respectively). This sensor performed best in the minimalist shoes, possibly because the minimal cushioning meant that the peak acceleration under the foot closely matched the vertical GRF loading rate. It is also possible that the fit of the shoe played a role, with the increased room in the neutral and maximalist shoes allowing more movement artifact for the Plantiga IMU.

While not all sensors were strongly positively associated with AVLR or IMeasureU-Tibia, they did all demonstrate a narrow distribution (see Table 2). Even though tibia-mounted PPA and vertical GRF loading rates have been associated with running-related injury, there is still much debate regarding the causal relationship of these variables with running-related injury (Ceyssens et al., 2019). Alternate sensor locations may prove to be useful in the future to monitor impact-related metrics associated with injury risk or performance outcomes. Furthermore, the practicality of these sensor locations–for instance, in studies in which participants are expected to affix the IMUs themselves or when large numbers of participants are involved—may outweigh the utility of the tibia-mounted site in larger field-based studies, especially if the location produces a more reliable output. Even when researchers have affixed tibia-mounted IMUs themselves with great care, there has been a large range of correlations to vertical GRF loading rates (Hennig et al., 1993; Laughton et al., 2003; Van den Berghe et al., 2019; Tenforde et al., 2020). One potential method to improve results from IMUs is by applying artificial intelligence to large data sets that include gold standard force treadmill/force plate data as well as IMU data. As has been demonstrated recently, the use of machine learning algorithms may further improve the estimation of GRF variables using accelerometer inputs (Ngoh et al., 2018; Jiang et al., 2020). More research in this area is warranted.

There are some limitations to this study. We were unable to synchronize the IMUs with each other or with force data from the treadmill. While we are confident that the between-step variability in all measures was low when averaged over 30 steps on the treadmill, this method could have negatively influenced the associations in some instances. Our a priori sample size calculation was based on previous studies' findings of correlations between tibia-mounted PPA and AVLR (Hennig et al., 1993; Laughton et al., 2003; Van den Berghe et al., 2019; Tenforde et al., 2020). However, given some of the lower correlations in different conditions, as well as the lower correlation at different sensor locations, the study turned out to be slightly underpowered. The small sample size resulted in underpowered estimates and large confidence intervals in individual conditions, which made significant findings between conditions difficult to achieve. Our sample comprised only rearfoot strikers, meaning that our findings should not be extrapolated to non-rearfoot striking runners.

## Conclusion

Our findings indicate that a commercially available insole-embedded IMU (Plantiga) is comparable to a research-grade tibia-mounted IMU (IMeasureU) when acting as a surrogate for vertical ground reaction force loading rates. However, these results vary between different levels of footwear and running speeds. A shoe-mounted RunScribe IMU exhibited slightly lower positive associations with vertical ground reaction force loading rates. In general, the Plantiga IMU performed better at faster running speeds and in minimalist footwear, while the RunScribe IMU performed better at slower running speeds and in the more cushioned footwear conditions. However, both the RunScribe and Plantiga IMU demonstrated only low positive associations with the tibia-mounted IMeasureU sensor, and this varied significantly across both footwear and speed conditions. Further investigations into the effect of footwear and location of IMU when measuring PPA are warranted.

## Data Availability Statement

The raw data supporting the conclusions of this article will be made available by the authors, without undue reservation.

## Ethics Statement

The studies involving human participants were reviewed and approved by Simon Fraser University Research Ethics Board. The patients/participants provided their written informed consent to participate in this study.

## Author Contributions

CN, CM, and RW designed the study, reviewed, and edited the final manuscript. CN carried out all data collection. CN, RW, BH, and RM were involved in data analysis. CN drafted the initial manuscript. All authors were involved in the interpretation and discussion of the results.

## Conflict of Interest

The authors declare that the research was conducted in the absence of any commercial or financial relationships that could be construed as a potential conflict of interest.
